# Healthcare professionals’ and parents’ experiences of the confirmatory testing period: a qualitative study of the UK expanded newborn screening pilot

**DOI:** 10.1186/s12887-017-0873-1

**Published:** 2017-05-08

**Authors:** Louise Moody, Lou Atkinson, Isher Kehal, James R. Bonham

**Affiliations:** 1 School of Art and Design, Coventry University, Priory Street, Coventry, CV1 5FB UK; 2 Centre for Applied Biological & Exercise Sciences, Coventry University, Coventry, CV1 5FB UK; 3 Centre for Technology Enabled Health Research, Coventry University, Coventry, CV1 5FB UK; 4 Newborn Screening Laboratory, Sheffield Children’s NHS Foundation Trust, Western Bank, Sheffield, S10 2TH UK

**Keywords:** Expanded newborn screening, Confirmatory testing, Parents experiences, HCPs experiences, Communication of screening results

## Abstract

**Background:**

With further expansion of the number of conditions for which newborn screening can be undertaken, it is timely to consider the impact of positive screening results and the confirmatory testing period on the families involved. This study was undertaken as part of a larger programme of work to evaluate the Expanded Newborn Screening (ENBS) programme in the United Kingdom (UK). It was aimed to determine the views and experiences of healthcare professionals (HCPs) and parents on communication and interaction during the period of confirmatory testing following a positive screening result.

**Methods:**

Semi-structured interviews were undertaken with parents of children who had received a positive ENBS result and HCPs who had been involved with the diagnosis and support of parents. Ten parents and 11 healthcare professionals took part in the in-depth interviews. Questions considered the journey from the positive screening result through confirmatory testing to a confirmed diagnosis and the communication and interaction between the parents and HCPs that they had been experienced. Key themes were identified through thematic analysis.

**Results:**

The results point to a number of elements within the path through confirmatory testing that are difficult for parents and could be further developed to improve the experience. These include the way in which the results are communicated to parents, rapid turnaround of results, offering a consistent approach, exploring interventions to support family relationships and reviewing the workload and scheduling implications for healthcare professionals.

**Conclusions:**

As technology enables newborn screening of a larger number of conditions, there is an increasing need to consider and mediate the potentially negative effects on families. The findings from this study point to a number of elements within the path through confirmatory testing that are difficult for parents and could be further developed to benefit the family experience.

**Electronic supplementary material:**

The online version of this article (doi:10.1186/s12887-017-0873-1) contains supplementary material, which is available to authorized users.

## Background

The advantages of newborn screening (NBS) in terms of early detection and treatment of serious conditions are well-documented [1–3]. Until recently in the UK routine newborn screening (NBS) was undertaken for five conditions Sickle cell disease, Cystic Fibrosis, Congenital Hypothyroidism, Phenylketonuria and Medium-Chain Acyl-CoA Dehydrogenase Deficiency (MCADD) [4]. In 2012 Expanded newborn screening (ENBS) for five additional inherited metabolic diseases (IMDs) was introduced as a pilot programme for Maple Syrup Urine Disease (MSUD), Homocystinuria (pyridoxine unresponsive) (HCU), Isovaleric Acidaemia (IVA), Glutaric Aciduria Type 1 (GA1) and Long Chain Hydroxyl Acyl CoA Dehydrogenase Deficiency (LCHADD) at six centres in England [4–6]. From 2015, the UK National Screening Committee adopted screening for four of the five additional conditions (HCU, MSUD, GA1 and IVA) within the UK NBS programme.

With the expansion of screening programmes to include additional rare conditions, there is a need to further consider, and minimise the impact on families where possible [7, 8]. Expanded screening will increase the identifications of conditions (true positives), but also result in an increase in the number of false positive results where an initial out of range screening result for a condition is followed by confirmatory testing that indicates the disorder is not present [8–11]. This lower positive predictive value associated with a screen positive test result is characteristic of some of the newer candidate disorders. Whether a condition is found to be present, the period of confirmatory testing can cause significant anxiety for the families concerned as they wait for results [12–14]. Research has indicated an impact on family relationships, parental depression and ongoing relationships with health care professionals (HCPs) [15, 16]. The communication and support provided during the confirmatory testing period are thought to mediate the impact on the family [9, 13, 17–20].

Guidelines exist to guide the communication of screening results [16, 21, 22] but implementation is believed to vary in practise and further exploration of parental and clinician views is warranted. Existing research on communication in this context has been conducted mainly in the USA and findings cannot be directly applied to the UK where screening is designed as a community based activity with an emphasis on integrated care during the maternity pathway [23].

The study described here was undertaken as part of a larger programme of work to evaluate the ENBS pilot in the UK. ENBS pilot studies have tended to report on the performance of the service (e.g. screen-positive prevalence and predictive value, screening uptake) [24–26] with limited exploration of communication and the parental and clinician experience during the pilot. Here it was aimed specifically to determine the views and experiences of healthcare professionals (HCPs) and parents on communication and interaction during the period of confirmatory testing following a positive screening result.

## Methods

Semi-structured interviews were undertaken with parents of children who had received a positive ENBS result and HCPs who had been involved with the diagnosis and support of parents during the pilot period (July 2012–July 2013). The study was approved by the National Research Ethics Service (East Midlands committee, Northampton, UK). All participants gave their written informed consent prior to participation in the study.

### Recruitment and participants

The ENBS program involved screening across six centres in the UK: Sheffield Children’s NHS Foundation Trust, Leeds, Great Ormond Street Hospital, Manchester Children’s Hospital, Birmingham Children’s Hospital and Guy’s and St Thomas’ NHS Foundation Trust. Inclusion criteria for the HCP sample stated that Metabolic Physicians, Specialist Metabolic Nurses, and Specialist Metabolic Dieticians involved in the ENBS pilot with experience of communicating screening results to parents would be invited to take part. A key factor for inclusion was availability for interview, as getting busy clinical staff for interview was an issue, and therefore there was an element of opportunity involved. Eleven semi-structured interviews were conducted with six metabolic physicians, two nurses and three dieticians. This represented at least one member of staff from each of the six screening centres.

The parental sample included mothers and fathers who received a true or a false positive ENBS result and were able to give informed consent. Parents under 16 or unable to give informed consent were excluded. During the ENBS pilot there were total of 30 screen positives (12 false positives and 18 true positive). They were asked by the NHS Trusts whether they would consent to being contacted about related research. Seventeen provided consent to be contacted about the study; nine of these were successfully contacted by telephone and agreed to take part. To improve recruitment, the sample was later widened to include those who were screened for these conditions while screening was extended and one additional participant recruited.

Ten parents took part; two interviews were undertaken as paired interviews with both the father and mother present. Two participants were recruited through Great Ormond Street, one from Sheffield, one from Leeds and six from Birmingham. The conditions for which they screened positive, and time elapsed between the screening result and the interview are indicated in Table 1. The parents and HCPs involved were not matched in this study; whilst the screening centre for each parental participant was recorded, the HCPs involved in the process were not.

**Table 1:** Summary of participant demographics

Gender of participant	Age of child at interview	Condition	True (TP) or false positive (FP) screen
Male	21 months	GA1	TP
Female	24 months	GA1	TP
Female	9 months	GA1	TP
Female	23 months	IVA	FP –diagnosis of benign condition
Female	17 months	HCU	FP –equivocal results
Female	24 months	MSUD	TP
Female Male	24 month	IVA	TP
Male Female	14 months	HCU	TP

Eight participants’ children had a true positive screen. Two were identified as false positive cases; however further testing led to a diagnosis of a benign condition in one case, and equivocal results for the other as reported by the parents at the time of the interview. Four out of the five ENBS conditions were represented. The children ranged from being healthy, managed through diet, to having been very sick at discrete episodes.

### Procedure

The semi-structured interview schedules were developed through consultation with the project team (see Additional files 1 and 2 for interview schedules). For the HCPs, questions probed experiences of giving screening results, parental responses to the news, and their recommendations regarding communication. The differences in approach taken to parents suspected of having a true versus a false positive screening result were explored. Questions for the parents considered their journey from the positive screening result through confirmatory testing to a confirmed diagnosis and the communication and interaction with HCPs that they experienced. Whilst the approach offered some structure, it was possible to discuss issues as they were raised by participants.

The interviews were undertaken by three experienced researchers from Coventry University who have conducted many interviews within a healthcare context. They were unknown to the participants. The participants were briefed on the purpose of the interview and the researchers motivation to improve family experiences when the interviews were arranged. The HCP interviews were either undertaken face to face at the NHS Trust sites, or on the telephone to suit the requirements of the participants. The interviews lasted 30–60-min. The interviews with parents lasted 20–60 min and took place in the homes of the parents or over the telephone to suit the participant. Two interviews involved the parents being interviewed together. Typically a young child was present too. One interview was undertaken in a combination of Urdu and Punjabi, and another in Arabic, these were translated during transcription.

### Analysis

The interviews were recorded, transcribed verbatim and analysed independently by two researchers using thematic content analysis to identify patterns within the data [27]. Transcripts were coded line by line to identify relevant aspects of the data, once a comprehensive set of codes had been identified; repeated patterns across the data set were identified to generate themes. These themes were then reviewed, refined and illustrative quotations selected.

### Results

HCP and parent viewpoints have been synthesised together in identifying key themes and emerging recommendations summarised in Table 2.

**Table 2:** Recommendations for improving communication and interaction

A summary of emerging recommendations and areas for future research
Pre-screening • Awareness raising of the ENBS conditions amongst parents, the public and wider HCP community.
Initial contact • Development of exemplar communication scripts for the first call to parents to relay screening results and / or arrange an appointment; co-design of content with parents and HCPs. • Ensure direct contact between a specialist and the family the same day the parents are notified of the positive screening result • Ensure access to advice and support during the period spent waiting for a meeting with a specialist. • Development of information related to true and false positive results to be received by parents at the point of an initial screening result being relayed; co-design of content with parents and HCPs. • Ensure the availability of a neutral translator for relaying the information to parents whose first language is not English.
Waiting for a confirmatory diagnosis • Rapid turnaround of confirmatory results, ensuring that accurate timeframes are given to the parents. • Ensure access to support systems during the waiting period. • Provision of clear, actionable information in verbal, written and online mobile formats that can be easily shared. • Provide clear guidance and maternal support around breastfeeding. • Encourage personal support from a friend or wider family especially if there is only one parent available.
Long-term support • Ensure availability and referral to psychological support where required once a diagnosis is reached. • Development of a toolkit that provides condition specific information for non-specialist clinicians to provide support to families from the screening test onwards. • Development of an online self-management resource for parents that provides condition specific information and support for parents from screening through to ongoing condition management.
System support mechanisms • Consider how a consistent approach and service can be offered to support families and embed evidence-based guidelines whilst taking into account local context and resources. • Review the resource implications of screen positives and conditions identified through screening, to make recommendations regarding resource allocation. • Consideration of adaptable workload models and scheduling to cater for the management of screen positive cases whilst minimising the impact on the HCPs involved.
Future research • Explore factors affecting diagnostic acceptance and how these factors can be affected by clinical interactions. • Explore psychological interventions to support family relationships whilst managing a child with a long-term condition.

### Pre-screening information

Parents recalled consenting to take part in the ENBS pilot programme [6] but felt they had little or no knowledge of the ENBS condition with which their child received a positive screen:

*“He’s got a condition that we’ve not heard of before” (P1: L230).*

Parents had little recollection of pre-screening information and indicated there was a need for more general awareness of the conditions. They reported that they had not expected to hear back following the ‘heel-prick’ and so felt unprepared for the result. HCPs agreed that in their experience, parents typically had not heard of the ENBS conditions and were unfamiliar with the available pre-screening information.

### Initial contact

The first contact between the parent and an HCP to relay the screening result left a strong lasting memory with parents. Contact was made by telephone either to arrange a home visit by the midwife or a nurse, or to ask the parents to go into the hospital (with variations per area). The anxiety and long term memory of this initial call was recognised by both parents and HCPs:

*“..the very first phone call that they receive, that information of a positive and there could be something, that is the one that remains with them, how that has been dealt with, how it’s been communicated, that is the important one” (HCP5: L46–8).*

*“It just sends you into panic……It makes you feel really sick doesn’t it (P7: L448–451).*

Parents recommended that the initial phone call needed to provide ‘the right amount of information’ but the quantity and content was difficult to define:

*“I think when they ring you up they should explain like what is it, they should tell you straight on the phone why, because why should you be worried and getting stressed, crying because, or your baby might die, or it might be serious..” (P3: L116–119).*

*“I would try and avoid if it was me. I would try and avoid giving away too much detail until you’re in a position sat talking to these people where you can explain a lot more fully rather than just this horrible tit-bit of information.” (HCP8 L466–469).*

This initial contact was perceived to affect anxiety during the first face to face meeting with the consultant specialist. There was agreement between parents and HCPs that further consideration of the best way to make contact with parents would be beneficial.

In some regions a screening or metabolic nurse was able to go out to the family with the midwife to relay the news of the positive screening result providing a familiar HCP and a subject specialist, but was not always possible due to resource limitations. It was agreed that direct contact between the HCP and family the same day was important. The reported time between the phone call and direct contact with the specialist team varied from about 15 min to 2 days. Parents felt, where there was a delay some contact was needed with a knowledgeable professional:

*“… I suppose if somebody had come round and sat down with us to try and keep us a bit calmer cos to have a few minute phone call and then put the phone down and sent an email you think Jesus Christ this is awful” (P2: L226–229).*

### First consultation

The initial consultation between the parents, the metabolic specialist and their team was typically a long meeting, lasting 1 to 4 h. Typically a multi-professional team approach (including a registrar, a screening and or metabolic nurse and dietician) was taken to facilitate parental and HCP understanding.

HCPs indicated that they give core information about the condition and testing process, and then tailor their approach and further information to the family needs and understanding. The conditions are rare, hard to understand and usually unknown to the parents:

*“..the conditions they’re not names that people are familiar with. I had one parent say to me ‘You know if you told me it was down-syndrome I could understand’, so we then have the other problem of this huge strange name that people can’t even pronounce, so I kind of break it down to things, sizeable information bits that they can comprehend and then tell them not to bother with this huge name but that’s what it means.” (P5: L113–8).*

Some parents were reported by HCPs to take the news badly find the news of the screening result very difficult, or not take it in. In general, parents indicated that at this time they had tended to focus on the future, the worst case scenario, and the implications of the condition and wanted an accurate assessment of the likely reality:

*“I just wanted to know for my child what is going to happen to her…“(P2:L160).*

Parents recalled being given an indication of the likely diagnosis at this stage:

*“…they said the heel prick was really high so it’s more than likely that he’s got it “(P3 L284–5).*

*“Within half an hour of speaking to us they had kind of…they only told us that something might be horribly wrong but also put our minds at rest by saying it’s odd that you know they would have expected to see symptoms” (P8: L58–60).*

HCPS were asked if they adapted their style if they suspected a diagnosis would be reached from the positive screening result. The majority of the metabolic consultants felt they would adapt their communication style if the screening marker levels strongly indicated a true or false positive to minimise parental anxiety:

*“Now, normally you’ve got some inkling of which direction this is going to be, so again, you should have tried to prepare them for the result you think you’re going to get. And you know, 90% of the time you’re probably fairly accurate in that (P6: L152–4).*

Some cases were considered easy to clarify (for example premature or ill babies), and were managed in such a way to avoid causing too much anxiety. More confidence in the likely outcome evolved over the ENBS pilot.

Typically (as reported by parents and the HCPs) both the mother and father were able to attend the appointments, and sometimes an additional family member too. HCPs believed that it was preferable for the parent to have someone else present for emotional support, to inform discussions about the parental history for inherited conditions, and ensure the family has the knowledge to manage the child. However, it was also noted that it can be difficult if there are too many people in the room (including HCPs, family and interpreters):

*“my concern is getting the balance right between too many people in the room because that can actually be difficult sometimes, it’s a bit overwhelming for parents...” (HCP8 L287–9).*

The HCPs and parents agreed that the first face to face contact and consultation with a specialist led to reassurance:

*“So most parents will cry at the end of the clinic or afterwards when they’re with the nurses or dieticians, but actually they normally leave feeling much better than they did when they came”.(HCP6 L85–7).*

*“Once we got to the hospital they were knowledgeable and professional – just the wait and initial call not good” (P7 L497).*

However, it was recognised by a number of parents that they were too anxious during the first consultation to take in all of the information given:

*“Yeah probably just you know all little charts and things what does that mean and what’s this and genes are missing here and I don’t know, I don’t know what you’re talking about and at the time I can’t process because I’m just worried and upset for my baby and yeah it’s too much”. (P2 L175–8).*

The HCPs indicated that they aim to strike a balance between the reality of the situation, worrying the parents unnecessarily, and setting them at ease and therefore try to keep information simple and spread out over the day.

### Waiting for a confirmatory diagnosis

The period of waiting for confirmatory results was reported to vary between hours to a week depending on the condition and treatment protocol. For some conditions families will go home as a day case to await confirmatory results. For others (e.g. LCHADD) children were admitted to the ward. HCPs noted that this can be difficult for the parents, but gives the clinicians the opportunity to spend time with the family, explain the condition and help with dietary management whilst monitoring the child.

HCPs felt that the nature of the symptoms (e.g. sleepiness, vomiting) for some ENBS conditions can be hard to assess in a newborn which can lead to parental anxiety and trips to hospital during this period. Waiting for confirmatory results was acknowledged as difficult by HCPs, but considered better managed when parents are reassured and have the information they need.

Parents reported that waiting for confirmatory results was stressful. They believed that the hospital had tried to turn the result around as quickly as possible:

*“..think they did it as quickly as they could so that was quite reassuring cos it was ...you just want to know don’t you so they were quite good like that so yeah they did it as quickly as they could, I don’t think they could have done much better than they did”. (P8 L479–482).*

Whilst waiting, some parents focused on the screening result being inaccurate, others assumed the worst case scenario:

*“And I was like wow you’ve pretty much confirmed that she’s got it, but to say you know 99% but we have to do another one to set it to stone to confirm it properly… but then I kept holding on to that 1% thinking there’s still that chance”.(P2: L185–187.*

*“No, cause when you’re finding out that your baby can end up like a flower and not moving at all, have brain damage and I don’t think anybody would be not stressed.” (P3: L311–2).*

Some parents needed to implement dietary changes during the waiting period (condition dependent). The ability to breastfeed and accurate dietetics information about this was recalled as important to mothers. One mother reported that the stress stopped her from being able to breastfeed.

### Reaching a diagnosis

HCPs indicated that where possible the news of a confirmatory diagnosis was given face to face, and where given on the telephone, the parents were called in to discuss. Some parents reported receiving results by telephone and some through face to face appointment. The parental response to a diagnosis was described by the HCPs as individual and variable but often reported to be one of great upset:

*“Without a doubt there’s just going to be floods of tears, shock and everything” (HCP5: L399).*

*“…it is bad news you can’t pretend it’s not you know for families, you’re telling them that their child has a lifelong condition that requires management.” (HCP3;L265–7).*

There was less detail recalled by parents about the relaying of the confirmatory diagnosis than the initial call about the screening result:

*“So we must have gone four times in the first year but I think, I don’t know about getting the diagnosis.” (P8; 209–10).*

The conditions are complex, and parents felt it took time to absorb the information that their child’s condition was confirmed:

*“It took a long time to absorb it and understand what was wrong with her…It was very overwhelming, and just like wow, and you know at first I thought oh my gosh this is awful” (P2: L67–72),*

HCPs reported parents asking questions about treatment, how the child would be affected, how often they would be seen; alongside concern about the future quality of life. Only one parent reported in the interview feeling that she was unprepared for the reality of her child’s condition. The majority felt they were given appropriate information and support and felt reassured once treatment was underway.

HCPs report that they aimed to communicate that the screening and confirmatory test result was a good day (not a bad one) as the knowledge of the condition would improve child health. They noted cases of parental denial of the condition where the child is not visibly unwell; or in other cases parents had prepared themselves for the worst case scenario.

HCPs were asked about the parental response to a false positive screening result. It was typically described as relief. In the majority of cases the parents were reported not to make contact again, but some had rung for reassurance. The management of the conditions vary and so may affect the impact of the false positive on the family. HCP contact with these families is not maintained so an ongoing impact may not be evident, but it was hoped that long term impact could be moderated through the cases being well managed.

The parental sample included two families who had a longer period of diagnostic uncertainty. The screening had detected a raised metabolite but had not led to diagnosis of a screened for condition. In one case a clear diagnosis was still not available to the parents at the time of the interview; in the other a mild variant of further condition was diagnosed. In both cases the parents were glad the issue has been identified through ENBS, explored and the child monitored.

### Longer term family impact

Parents’ longer term responses to the diagnosis (as described at the time of the interview) varied:

*“..but she’s not a normal child, she’s not normal. If she was normal I wouldn’t worry as much” (P4: 180–1).*

*“I am happy that my baby is now normal like other children, Her food is different…..I am very happy, the whole credit goes to the metabolic team “(P6 L244–6).*

One mother explained the conflict between wanting to protect her child versus the desire to treat her normally. Another indicated that she found it hard to explain to her family what was wrong with the child and why she worries.

The parents felt that HCP support since diagnosis was knowledgeable and well managed:

*“P7: They seemed very knowledgeable….*

*P8: …I don’t think there was a single ‘oh I’ll have to come back to you’ answer to any of our questions, I think everything was just answered there and then, like I say we were dealing with people that…..” (P7 & 8: L284–289).*

Family support had been important. The challenge of managing frequent hospital trips alongside care of other children and employment was highlighted. Some mothers had taken over management of the child’s condition and family relationships were affected:

*“To be very frank, the biggest difference was on my personal life. Because what a mum can do, a father can’t do. I am to this day doing it for my baby. Other family friends, have also been affected, I can’t get out as much” (PT6: L209–11).*

*“.... cos I’m always stressed over her, like I do everything for her and I run around her quite a lot (PT4: L193-4)”.*

Three of the mothers interviewed indicated that their relationships had broken down and they linked the demands of having a child living with a condition. The diagnosis also raised questions for them around genetic carriers within the wider family and the impact on future children.

### Information resources and support mechanisms

The approach to communication by clinicians was protocol driven, but styles were recognised to vary. HCPs felt that communication and interaction with parents during the confirmatory testing period should be honest and reassuring, and some felt it was importance to demonstrate confidence. The importance of breaking down information to meet parental needs and using an interpreter for parents with limited English was highlighted by HCPs and non-English speaking parents.

Standard ENBS information sheets, emergency letters, and follow up information were used across the centres. The ENBS pilot website provided condition specific information as well as videos, and charity links etc. Parents generally reported that they received clear and well explained information that helped them explain the condition to family members:

*“yeah, we, you know what, early we had so much information on that condition from them and even from the website, so all we wanted, we did get the information”. (PT1: L216–8).*

Some HCPs recommended the website to deter parents from finding factually incorrect information online. Others waited to refer to the website until a condition was confirmed or warned parents off the internet due to the tendency to find ‘worst case scenario’ cases on unapproved sites. The majority of parents indicated that they had sought out information online.

The screening centres provided direct telephone numbers for support and advice from the specialist team. The contact numbers were often used by parents once diagnosis was confirmed, and sometimes whilst waiting for confirmatory results:

*“I felt reassured because as soon as there’s a problem we’ve got a number for them to ring them straight away you know, any questions or anything we’ve got a direct number to them so”. (PT5 L180–20).*

The value of support groups was explored. It was felt to be of mixed value amongst HCPs depending on the nature and attendees of the group. Caution was expressed that larger support groups could lead parents to be scared by different experiences. One mother wanted support to manage the demands of the condition. An interest was expressed in informal 1:1 relationships between families affected in a similar ways by the same condition.

### Impact of ENBS

Parents felt lucky that they had benefited from the pilot programme and were grateful to have been provided the opportunity for early treatment. Only one of the interviewed parents found that the uncertainty around the condition and its early treatment had led to mistrust in the treatment and HCPs.

Parents felt that there is limited awareness of ENBS conditions and a need for increased knowledge amongst the public and doctors in local hospitals:

*“Yeah and even my doctor, my doctors been a doctor for 30 odd years and the doctor at our local hospital has been a doctor for 40 years and they’ve never heard of it and I’m thinking I’ve now got to educate you guys (PT2: L69-71).”*

From the HCP perspective, ENBS has led to new knowledge and earlier diagnosis. HCPs reported initially relying on their experience with existing screened conditions, but condition specific knowledge and approaches developed over the pilot. The learning curve was recognised for managing cases as well as communication with the family.

HCPs were asked about the impact of ENBS on themselves. The stress and anxiety involved in screening was noted by some HCPs both in terms of the emotional management of families but also due to the workload implications. A positive screen takes priority which can be hard to manage alongside other work demands, given the resources needed to reach a diagnosis.

*“Even the positives are additional but people say it’s not, but the thing is actually it’s hard to even talk to the Managers and say it’s an ad-hoc thing, whenever it comes positive I have to drop everything” (HCP5: L:450).*

For true positive screening outcomes, there is a grieving process for the parents to be managed; as well as the treatment plan and ongoing consultation.

## Discussion

The expansion of screening to include additional complex and rare conditions presents challenges in terms of providing accessible information to parents, and communicating results effectively through sensitive and supportive interactions with HCPs. This study explored parental and HCP experiences of the UK ENBS pilot with a focus on the period of confirmatory testing following a positive screening result. The views of the parents and HCPs tended to be in alignment raising similar issues of concern.

Awareness of the ENBS conditions was reported to be low amongst parents prior to receiving the screening results. Given the careful design of the ENBS information and consent model prior to the pilot [6], it is likely that parents did receive information and were explained the nature of the pilot. However, in line with previous research they may not have fully attended to, or retained the condition specific information provided [28, 29]. As providing adequate information for rare and complex conditions continues to be a challenge, it is argued that co-design [30] of paper-based and online information with parents may further enhance the accessibility of the key messages. Broader awareness raising of ENBS conditions amongst the public and wider HCP community may also be useful to aid parental and wider family understanding.

The first contact, often by telephone, with parents following a positive screening result leads to anxiety and lasting memories for parents whilst being acknowledged as particularly difficult by HCPs. The content of the information provided ahead of a face to face meeting warrants further consideration, particularly in areas of the country where there may be a delay before direct contact with a specialist. Communication scripts co-developed with parents and HCPs are suggested as a possible means to guiding the first contact.

The rapid availability of reliable and accessible condition specific information as well as support from HCPs and family is important. In line with the literature, participants’ views suggested that early direct contact between the specialist team and the family and continued support, is likely to affect the long-term impact on the family [21, 31]. Parents clearly have a desire to seek information online and the availability of credible, reliable resources that reflect the early detection of conditions and discourage parents from accessing negative images and case studies is important. Condition specific information can be overwhelming and hard to process at a time of high anxiety for parents, so content should be simple, clear and actionable and where possible tailored to individual needs. The presence of an independent translator where required to ensure that information can be processed and responded to by the parent is an imperative.

The increase in positive screening results (both true and false positives) through ENBS, places a new and different workload on HCPs and the healthcare system. There was variation evident during the pilot due to local resources and circumstances that affected parental experience. It is important to consider how a consistent approach and service can be offered to embed evidence-based guidelines and protocols whilst taking into account local context and resources. An assessment of the resource implications of the confirmatory testing period alongside adaptable workload models and scheduling to cater for the management of screen positive cases is needed to support the HCPs involved.

HCPs drew attention to the need to rapidly develop healthcare knowledge to manage early detection of the ENBS conditions as well as to effectively communicate with and support families. The availability of online toolkits to support clinical knowledge development, and offer a self-management resource for parents that provides condition specific information and support from screening through to ongoing condition management may be mechanisms to help support cost-effective delivery.

### Study limitations and future research

The participant sample in this study was relatively small due to the inclusion criteria and challenge of recruitment. However, the interviews benefitted from an in-depth reflection on experiences and the HCPs interviewed could reflect on multiple cases that they had been associated with. Within the parental sample, participants’ children had been affected by different conditions, treated at different centres and received different diagnoses. Cases will have been managed differently; however, it is argued that it is this variability that is of interest.

The time elapsed between receipt of the screening result and the interview means the data is reliant on recollection. However, it also reflects the longer-term view of the parents and the impact on them. It provides a useful collection of views about the emotional response to the experience, although it is accepted that factual data about information provided e.g. time frames form parents may not be accurate.

Men are under-represented in research of this nature. Fathers would often be on paternity leave at the time of receiving the screening result; but accessing men for the interviews sometime later proved difficult. Given the impact on family relationships and employability, their continued involvement in screening research is particularly important.

The study highlighted varying responses to the diagnostic experience. Future research is needed into the factors affecting diagnostic acceptance and the impact of clinical interactions on the long-term family response to managing the conditions. As understanding increases of the impact of screening and managing a child’s IMD, it would be valuable to explore psychological interventions to guide parents bonding and maintaining family relationships to help families to address the daily challenges they may face.

## Conclusions

This study sought to understand and learn from participants’ experiences of an ENBS pilot in the UK. With further expansion of the number of conditions for which screening can be undertaken and the increased rate of false positive results observed in some of the newly included disorders, it is timely to consider how the impact of positive screening results can be minimised. Although there are studies exploring the impact of receiving positive NBS results, many focus on quantifying the impact rather than detailed exploration of experiences.

This qualitative study of experiences has highlighted elements within the path through confirmatory testing that are difficult for parents and could be further developed to benefit the family experience. In particularly, the findings recognise there is a need to further develop the information given and the mechanisms by which parents are communicated with and supported. Recommendations have been made to address some of the challenges raised by participants in order to further minimise the impact on families of positive screening results.

## Additional file

Additional file 1: Semi-structured interview schedule - Parents. (ODT 19 kb)
Additional file 2: Semi-structured interview schedule - Healthcare Professionals. (ODT 16 kb)

## Abbreviations

ENBS: Expanded Newborn Screening
GA1: Glutaric Aciduria Type 1
HCP: Healthcare professional
HCU: Homocystinuria (pyridoxine unresponsive)
IMD: Inherited Metabolic Disease
IVA: Isovaleric Acidaemia
LCHADD: Long Chain Hydroxyl Acyl CoA Dehydrogenase Deficiency
MSUD: Maple Syrup Urine Disease
NBS: Newborn screening
UK: United Kingdom
