# Supplementary material for: Healthcare professionals’ and parents’ experiences of the confirmatory testing period: a qualitative study of the UK expanded newborn screening pilot
Source: BMC Pediatr. 2017 May 8;17:121. doi: 10.1186/s12887-017-0873-1 (PMC5422997; doi:10.1186/s12887-017-0873-1)
Supplement: Supplementary file 1 [file 12887_2017_873_MOESM1_ESM.odt]

Semi-structured interview schedule - Parents 

| Demographic information Current age of the child Approximate date of screening Area of country (i.e. screening centre) Condition to which the result applies Final diagnosis (i.e. false or true positive) |
| --- |
| Interview prompts How well do you remember receiving your child’s initial (out of range) screening result and what happened next? How long was it before you received a confirmatory test result? How did you find this experience? How did you feel at the time? How effective was the communication and interaction (between the initial and confirmatory result) between you and the Doctor (hospital team) at this time? (Interviewer to differentiate the communication of the NBS result and the communication of the diagnosis, and anything in between where necessary for the participant) What information were you given? (written and verbal) Did you have enough information to fully understand the situation? How easy was the condition to understand? What did you understand about the likelihood of a confirmed diagnosis of [insert condition]? How did the information make you feel? E.g. reassured, worried, etc. Did you receive enough information, at the right time, in the right format? How might it be improved?  How about the people you spoke to? Did you trust them? Did they seem knowledgeable? Did they show empathy, was information delivered sensitively? How easy was it to communicate the situation to your partner and family?  Would additional support have been useful e.g. a helpline? If there is one, did you use it? Was it available 24 hrs / when needed?  What other support would you have liked? What was the impact on you and your family? How do you feel about receiving the result now? (interviewer to differentiate impact of NBS result, then impact of other diagnostic results) How well was the experience managed? Could you speak to someone when you needed to? How could the experience have been improved for you and your family? |
