# Supplementary material for: Healthcare professionals’ and parents’ experiences of the confirmatory testing period: a qualitative study of the UK expanded newborn screening pilot
Source: BMC Pediatr. 2017 May 8;17:121. doi: 10.1186/s12887-017-0873-1 (PMC5422997; doi:10.1186/s12887-017-0873-1)
Supplement: Supplementary file 2 [file 12887_2017_873_MOESM2_ESM.odt]

Semi-structured interview schedule -  Healthcare Professionals

| Demographic information   Position / role Approximate last date of last positive screening result Area of country from (i.e. screening centre / hospital) |
| --- |
| Interview prompts What has been your experience of managing positive (true and false) screening results?    What is the parental response typically in these situations?   In your experience, how effective was the communication and interaction in managing the parental response?    What impact did this experience have on you?   How do you think false positive test results should be handled? What works well? What doesn’t work well?   How do you manage parents differently for true positives and false positive screens?    How do the marker levels influence your interactions with the parents?    How much variation do you believe there is between individual clinicians’ management of the family as results are given?   What standardised information is given? Is this adequate / appropriate? How might it be improved? Do parents receive enough information, at the right time, in the right format?   Do you speak with both parents?   In your opinion would a helpline be useful?   How long typically before a confirmatory test result is given? How do parents respond to this delay?    What is the impact on the child and family of a false positive screening result in your experience?    How could the experience be improved? What would be your best practice recommendations? |
